# A Review of Current and Future Antithrombotic Strategies in Surgical Patients—Leaving the Graduated Compression Stockings Behind?

**DOI:** 10.3390/jcm10194294

**Published:** 2021-09-22

**Authors:** Amulya Khatri, Matthew Machin, Aditya Vijay, Safa Salim, Joseph Shalhoub, Alun Huw Davies

**Affiliations:** 1 Academic Section of Vascular Surgery, Department of Surgery & Cancer, Imperial College London, London W6 8RF, UK; amulya1@hotmail.co.uk (A.K.); matthew.machin12@imperial.ac.uk (M.M.); aditya.vijay15@imperial.ac.uk (A.V.); safa.salim11@imperial.ac.uk (S.S.); j.shalhoub@imperial.ac.uk (J.S.); 2 Imperial Vascular Unit, Imperial College Healthcare NHS Trust, Charing Cross Hospital, London W6 8RF, UK

**Keywords:** antithrombotics, anti-coagulants, anti-platelets, cardiovascular disease, ischaemic heart disease, atrial fibrillation, venous thromboembolism, thromboembolic risk prevention, bleeding risk management

## Abstract

Venous thromboembolism (VTE) remains an important consideration within surgery, with recent evidence looking to refine clinical guidance. This review provides a contemporary update of existing clinical evidence for antithrombotic regimens for surgical patients, providing future directions for prophylaxis regimens and research. For moderate to high VTE risk patients, existing evidence supports the use of heparins for prophylaxis. Direct oral anticoagulants (DOACs) have been validated within orthopaedic surgery, although there remain few completed randomised controlled trials in other surgical specialties. Recent trials have also cast doubt on the efficacy of mechanical prophylaxis, especially when adjuvant to pharmacological prophylaxis. Despite the ongoing uncertainty in higher VTE risk patients, there remains a lack of evidence for mechanical prophylaxis in low VTE risk patients, with a recent systematic search failing to identify high-quality evidence. Future research on rigorously developed and validated risk assessment models will allow the better stratification of patients for clinical and academic use. Mechanical prophylaxis’ role in modern practice remains uncertain, requiring high-quality trials to investigate select populations in which it may hold benefit and to explore whether intermittent pneumatic compression is more effective. The validation of DOACs and aspirin in wider specialties may permit pharmacological thromboprophylactic regimens that are easier to administer.

## 1. Introduction

Venous thromboembolism (VTE), an umbrella term encompassing deep venous thrombosis (DVT) and pulmonary embolism (PE), still represents a major cause of morbidity and mortality within the United Kingdom (UK) [1]. An astounding 20 to 50% of DVT cases go on to develop post-thrombotic syndrome due to venous insufficiency, resulting in leg oedema, pain, and impaired mobility [2]. VTE events also cause a significant economic impact, with an estimated cost of £640 million per annum when considering both hospital and community care [3].

Hospital-associated thrombosis, defined by VTE occurring within 90 days of hospital admission, is still relatively common with 60.4 cases per 100,000 hospital admissions in 2020 within the National Health Service (NHS) in England, failing nine cases per 100,000 in the last decade [1,4]. Surgery is a known risk factor for VTE, with major surgical procedures and orthopedic surgery known to be particularly high risk (odds ratio (OR) > 10), which is contrasted with laparoscopic surgery having a smaller risk (OR < 2) [5].

Since the Health Select Committee’s 2005 report on the rate of VTE incidence within the UK, the prevention of VTE around the time of surgery has dramatically improved. Mechanical and pharmacological interventions remain the two key paradigms in VTE prevention. Mechanical thromboprophylaxis largely consists of either graduated compression stockings (GCS) or sequential compression devices, such as intermittent pneumatic compression (IPC) devices, whereas pharmacological intervention typically involves low-molecular weight heparins (LMWH) or direct oral anticoagulants (DOAC).

In addition, wider systematic changes to the peri and post-operative care have also reduced the risk of VTE. The introduction of the Enhanced Recovery After Surgery (ERAS) protocols, ranging from early ambulation to improved surgical and anaesthesia regimens, have contributed to a reduction in VTE occurrence [6]. In addition, the systematic use of Risk Assessment Models (RAM) within healthcare prompt patient assessment, raise awareness, and direct the prescription of appropriate prophylaxis in individuals who previously may have otherwise been overlooked.

Current National Institute for Health and Care Excellence (NICE) guidance, published in 2018, advises that the thromboprophylaxis regimen be based on the patient’s risk of VTE according to the Department of Health (DoH) RAM, which incorporates known risk factors of thromboprophylaxis and the type of procedure being undertaken [4].

However, current clinical guidance around VTE prevention in surgical patients does not entirely align with evidence from recent randomised-controlled trials nor existing evidence in the literature. We aim to provide a summary of the contemporary state of evidence in addition to suggesting future directions for clinical practice and clinical research.

## 2. Current Clinical Practice in the UK

Current practices are guided by the NICE guidelines NG89, published in 2018 [4]. Surgical patients within the UK are risk stratified upon admission, or as soon as possible, should clinical circumstance not permit it. Within the UK, NICE guidelines recommend the use of the DoH RAM for all patients admitted, with 95% of patients admitted to NHS hospitals being assessed for VTE risk [7].

Within the score, assessors screen for any risk factors listed. Should a patient have a single risk factor with no contraindications, thromboprophylaxis is prescribed, with the specific regimen tailored subjectively to patients’ risks of VTE and bleeding alongside individual specialty guidance. Within the guidelines, no preference is given to either GCS or IPC for mechanical thromboprophylaxis. Blanket mechanical thromboprophylaxis is currently recommended for abdominal, thoracic, spinal, and bariatric surgery, with recommendation to consider its use in cardiac, cranial, vascular, head and neck, elective joint replacement and ear, nose, and throat (ENT) surgery. Blanket pharmacological prophylaxis is recommended for several orthopaedic and bariatric procedures. Use beyond these fields is considered based on individual patient risk factors.

## 3. Risk Assessment Models

RAMs have been an attractive option for clinicians, potentially allowing for tailored regimens to patient’s individual risk but also highlighting patients who require prophylaxis. From an academic standpoint, they permit risk quantification, allowing better comparison between similar patient groups.

However, current RAMs do have limitations, as previously critiqued [8]. The DoH RAM, used within the UK, does not provide different weighting per risk factor, despite each risk factor having varying risk for VTE development. Furthermore, the score provides no indication on the severity of risk, requiring the assessor to use their subjective assessment, leading to potential variation from best practice.

Other tools, such as the Caprini and Padua RAMs, are used in other countries, but these were deemed to be of low or very low quality in a literature review by NICE [4]. Whilst these scores utilise a graded system and provide a risk estimate, previous literature found using such scores resulted in overtreatment of patients deemed at low risk of VTE and undertreated patients deemed at high risk [9].

It is surprising that the weighting of individual risks differs between various RAMs, perhaps representing that such models may not be generalisable to all patient populations. For example, the Padua RAM was developed within a single centre in Italy, and the Kucher score was derived from a group with 80% cancer patients, who have an intrinsically higher risk of VTE [10,11,12].

Furthermore, whilst several models are used in clinical practice, their validation and application to wider clinical practice varies (Table 1). The DoH model remains unvalidated despite more than a decade of use [8]. The Caprini model has been validated through various retrospective studies for several specialties, such as general, vascular, ENT, and urology surgery patients, as well as in critically ill surgical patients, where the score was able to discriminate patients’ risk of VTE development [13,14,15]. The Padua, Kucher, and Intermountain RAMs were externally validated within acutely ill medical inpatients in the United States, where the authors found the model discrimination to be poor (c-index range 0.58–0.64) and noted the limited training population used, such as largely cancer patients (Kucher), or single centre with a small sample size (Padua) [12]. The International Medical Prevention Registry on Venous Thromboembolism (IMPROVE) RAM was externally validated in 19,217 medical inpatients, where the authors noted good discrimination (Receiver Operating Characteristic Curve 0.70) and calibration [16].

## 4. Current Clinical Guidelines 

In 2012, the American College of Chest Physicians (ACCP) guidelines were published, dividing surgical patient groups into orthopaedic and non-orthopaedic groups, due to orthopaedic surgery being intrinsically more prothrombotic. Table 2a,b summarise the guidance for non-orthopaedic patients [17].

Within non-orthopaedic groups, an important distinction of ACCP guidelines compared to NICE guidelines is that patients are stratified in accordance with their risk, through either estimated percentage likelihood of VTE, or the Rogers (very low < 7, low 7–10, moderate > 10) or Caprini score (very low 0, low 1–2, moderate 3–4, high ≥ 5). 

In general and abdomino-pelvic surgery, those at, and above, a low risk of VTE are recommended mechanical prophylaxis, with IPC preferred over GCS. For those at, and above, a moderate risk of VTE, pharmacological prophylaxis using LMWH or low-dose unfractionated heparin is recommended, unless the patient is at a high risk of bleeding complications.

For orthopaedic patients, those undergoing total hip or knee arthroplasty and hip fracture surgery are recommended pharmacological prophylaxis, preferably LMWH, or IPC for 10 to 14 days [18]. Those undergoing major orthopaedic surgery are suggested to have both pharmacological prophylaxis up to 35 days and IPC during the hospital stay. For isolated lower-limb injury requiring immobilisation, no prophylaxis is advised.

In 2018, the European Society of Anaesthesiologists (ESA) expanded upon the ACCP guidelines by producing guidance for specific clinical scenarios [19]. Table 3 summarises their recommendations for day-case/short-stay surgeries [20]. As in the ACCP guidelines, they do recommend the use of the Caprini score alongside the risk of the surgical procedure in determining the thromboprophylaxis regimen, although no recommendations are made for patients or procedures at moderate risk of VTE. Despite being less effective in VTE prevention than LMWH, aspirin is suggested to have a role in low VTE risk orthopaedic patients due to its reduced risk of bleeding. For mechanical prophylaxis, the ESA advise GCS should be used in conjunction with pharmacological prophylaxis, and that IPC is preferred over GCS. They also suggest combined mechanical and pharmacological prophylaxis for those at a very high risk of VTE [21].

## 5. Current Evidence Base for Moderate and High VTE Risk Surgical Patients

Patients from a variety of surgical disciplines may be deemed to be at moderate to high risk of VTE. Whilst both forms of prophylaxis are historically known to reduce the chance of VTE compared to no intervention [22,23], the optimal regimen for specific patient groups remains uncertain.

Currently, general surgical procedures are considered to be moderate to high VTE risk events, should the operative and anaesthetic length be more than 90 minutes, the operation cause significant immobility, or the condition requiring surgery be associated with an acute inflammatory condition. Other patient-specific factors, such as obesity and cancer, may individually or cumulatively constitute to a moderate VTE risk or higher.

### 5.1. Pharmacological Prophylaxis

Given the increasing volume of day-case procedures and early mobilisation following modern surgery, pharmacological prophylaxis remains further pertinent, especially with the lower efficacy of mechanical prophylaxis [24], alongside its potential impedance of mobilisation with IPC. Whilst heparins have traditionally been used as the mainstay for pharmacological prophylaxis, DOACs have been increasingly researched given the ease of administration.

A number of trials have investigated DOACs in either total hip or total knee arthroplasty patients, validating its use within joint replacement. The RECORD 1 and RECORD 4 trials compared rivaroxaban to enoxaparin in total hip and knee arthroplasty patients respectively, finding rivaroxaban to be significantly more effective in VTE prevention, with a comparable risk of bleeding [25,26]. Similar findings were also found for apixaban within the ADVANCE-2 and ADVANCE-3 trials. Dabigatran was non-inferior to enoxaparin for VTE and bleeding risk in the RE-MODEL and RE-NOVATE trials [27,28,29,30].

Outside of orthopaedic surgery, there remains a paucity of high-quality evidence. In 2020, an RCT investigated the use of argatroban in laparoscopic gynaecological malignancy surgery, randomising 307 patients to either half-dose LMWH, full-dose LMWH, or argatroban for 28 days [31]. The authors noted the incidence of DVT (*n* = 2) to not be statistically different between the various groups (0% vs. 0% vs. 2.38%, respectively). However, the trials results are limited by low numbers of participants and by being powered for superficial venous thrombosis rather than DVT. Several trials have also been initiated in various other patient groups, including colorectal cancer surgery, cardiac surgery, and breast reconstruction surgery, which may validate DOAC’s use outside of orthopaedics [32,33,34].

Aspirin has also been hypothesised to provide similar VTE prophylaxis to LMWH, whilst being easier to administer, inexpensive, and affording a potentially lower bleeding risk [35]. The EPCAT-II trial randomised 3424 hip and knee arthroplasty patients to receive either a 35-day course of rivaroxaban or a 5-day course of rivaroxaban followed by 30 days of aspirin [36]. The extended aspirin course was found to be non-inferior to the rivaroxaban course for VTE prevention (0.64% vs. 0.70%, *p* < 0.001 for non-inferiority), with no significant difference in the incidence of major bleeding (0.47% vs. 0.29%, *p* = 0.42). Importantly, relatively few patients included were at a higher risk of VTE, potentially limiting these results to lower risk elective arthroplasty patients [35].

Historic trials have also investigated the use of aspirin for the entire duration of pharmacological prophylaxis. In 2000, the Prevention of pulmonary embolism and deep vein thrombosis with low dose aspirin: Pulmonary Embolism Prevention (PEP) trial randomised 13,356 hip fracture surgery patients from multiple countries to receive either aspirin or placebo, finding aspirin to reduce both DVT by 29% (*p* = 0.03) and PE by 43% (*p* = 0.002), without any significant rise in death from excess bleeding. However, post-operative transfusion was significantly higher in the aspirin group [37]. A meta-analysis of 13 RCTs comparing aspirin to other forms of pharmacological prophylaxis in total hip or knee arthroplasty showed no statistically significant difference in DVT and PE rates [38]. Importantly, the risk of bleeding was not significantly different. These findings remained the same in sub-group analyses by year of publication, types of anticoagulants used as comparators, and excluding trials that had a hybrid course of another anticoagulant and then aspirin.

Further evidence is awaited from the EPCAT-III and the Comparative Effectiveness of Pulmonary Embolism Prevention After Hip and Knee Replacement (PEPPER) trials, providing the latest evidence to determine the efficacy of aspirin as a standalone VTE pharmacological thromboprophylaxis intervention [39,40].

Pharmacological prophylaxis is contraindicated if the risk of bleeding is high, such as in spinal or neurosurgical patients. A meta-analysis of heparin prophylaxis in craniotomy patients estimated that if 1000 patients were treated with heparin, between nine and 18 symptomatic VTE events would be prevented, at a cost of seven patients experiencing intracranial haemorrhage [41]. Considering the potentially catastrophic effects of intracranial haemorrhage, only mechanical prophylaxis is currently advised in both ACCP and NICE guidelines [4,17].

### 5.2. Mechanical Prophylaxis

Recently, there is increasing evidence questioning the role of mechanical prophylaxis in the context of wider improvements to VTE prophylaxis, particularly when pharmacological prophylaxis is in place.

In 2015, a systematic search assessing the added benefit of GCS when pharmacological prophylaxis was in place found the evidence base to be poor. The authors noted significant heterogeneity prohibiting pooled meta-analysis, with only one RCT identified investigating this specific comparison [42]. This trial investigated 874 hip surgery patients, which failed to demonstrate any significant difference in VTE incidence when GCS was added to a five-to-nine-day course of fondaparinux (combined vs. fondaparinux only, 4.8% vs. 5.5%, OR 0.88, 95% Confidence Intervals (CI) 0.46–1.46, *p* = 0.69) [43].

The GAPS trial, a multi-centre UK trial randomising 1905 moderate to high VTE risk elective surgical patients, aimed to investigate this uncertainty [44]. The trial found that GCS did not significantly reduce the incidence of VTE when pharmacological prophylaxis was already in place (1.4% vs. 1.7%, *p* < 0.001 for non-inferiority), further suggesting that GCS has limited benefit in these patients. It is important to note that the GAPS trial noted a surprisingly low incidence of VTE in comparison to historic data. These findings may represent the global improvement in VTE prevention outside of mechanical and pharmacological intervention, thus confounding the assessment of the use of GCS in modern surgical practice. These findings were also replicated in a recent single centre propensity matched study, further suggesting that GCS may hold minimal benefit if pharmacological prophylaxis is used [45].

There may be several reasons explaining the apparent lack of benefit in recent evidence. First, the overall incidence of DVT is reducing. As such, any potential VTE risk reduction from GCS has a minimal absolute reduction of VTE incidence. This is particularly compounded when pharmacological prophylaxis is in place, further reducing the VTE incidence. Secondly, GCS are thought to work by improving venous blood flow through replicating the calf-muscle pump mechanism. Given that patients mobilise much earlier than in the past, any adjunctive venous flow return may not be clinically sufficient to prevent DVT in acute surgical patients.

Outside of surgery, further doubt has been cast on the efficacy of GCS. The CLOTS1 randomised 2518 acutely admitted stroke patients to either GCS or no intervention, finding no significant difference in the incidence of proximal DVT’s, despite previous research suggesting GCS’s efficacy [46]. Whilst these results question GCS, they may not necessarily apply to surgical patients. In contrary to surgical patients, stroke patients received GCS later on once their mobility is reduced, perhaps limiting the trial results to medical patients.

The role of IPC in adjunct to pharmacological prophylaxis has seen conflicting evidence. A Cochrane meta-analysis compared IPC, pharmacological prophylaxis, and a combination of both, identifying 22 trials in various surgical specialties, although mostly within orthopaedic surgery [47]. The authors found combined IPC and pharmacological prophylaxis to be more effective than each intervention individually. However, the more recent PREVENT trial investigated 2003 critically ill patients, finding that combined IPC and pharmacological prophylaxis had no significant benefit in proximal DVT incidence compared to pharmacological prophylaxis alone (3.9% vs. 4.2%, relative risk (RR) 0.93, 95% CI 0.60–1.44, *p* = 0.74) [48]. The discrepancy between past and recent data may, again, be due to the wider improvement in VTE prevention leading to underpowering of the study, though it continues to question the use of mechanical prophylaxis when pharmacological prophylaxis is already in place.

Conversely, the addition of pharmacological prophylaxis to IPC was not found to have a benefit in a recent trial [49]. In 2020, 448 patients undergoing laparoscopic surgery for gastric and colorectal malignancies within Japan were randomised, with no significant difference found for the total incidence of VTE (IPC vs. combined, 4.8% vs. 3.3%, *p* = 0.453). With regard to bleeding complications, 10 minor and one major were observed within the combined group (5.4%, 95% CI 3.1–9.5%), whereas none occurred within the IPC-only group. It is important to note that all VTE events were asymptomatic and were diagnosed by active screening as per the trial protocol.

For patients who have contraindications to pharmacological prophylaxis, mechanical prophylaxis currently remains the mainstay compared to no intervention. Whilst historically both GCS and IPC are thought to hold benefit in surgical patients, recent data have suggested IPC to be more efficacious. The CLOTS 1 and 3 studies investigated GCS and IPC compared to placebo in acutely admitted stroke patients, respectively [46,50]. IPC was found to reduce VTE, whereas GCS had no such benefit. Previous meta-analyses have also found IPC to be more effective in critically ill and medically unwell patients [51,52].

This finding may be explained by comparing the modalities of compression. Intermittent compression is more comparable to the lower limb muscle pump physiology, perhaps resulting in this clinical difference [53]. However, it is important to note that IPC hinders mobility in comparison to GCS, resulting in either increased care needs to remove and then reapply IPC or delayed patient mobilization.

Neuromuscular electrical stimulation (NMES) is a novel form of mechanical prophylaxis that is yet to enter widespread clinical use, given the popularity and availability of GCS and IPC. NMES uses electrical impulses to stimulate leg muscle contraction, thereby improving venous blood flow. In 2014, NICE performed a medical technology assessment approving the use of the device for those in which other forms of mechanical prophylaxis are not suitable [54]. A meta-analysis performed in 2018 noted the existing evidence from RCTs to be limited by poor trial quality and heterogeneity in design [55]. NMES was found to be effective in DVT reduction compared to no intervention (OR 0.29, 95% CI 0.13–0.65, *p* = 0.003), inferior to heparin (OR 2.00, 95% CI 1.13–3.52, *p* = 0.02), and no added benefit when used in addition to heparin therapy (OR 0.33, 95% CI 0.10–1.14, *p* = 0.08). A subsequent Cochrane review also found NMES to reduce the risk of total VTE compared to no prophylaxis, although it was noted that there was no difference for symptomatic DVT nor PE and the evidence was also of low quality [56].

## 6. Current Evidence Base for Low VTE Risk Surgical Patients

Limited evidence exists for VTE prophylaxis regimens within low VTE risk surgical patients. Pharmacological prophylaxis use is not advised by the ACCP guidelines, as the risk of non-fatal major bleeding with LMWH (24 per 1000) is higher than the risk of non-fatal symptomatic VTE (five per 1000), whilst the risk of fatal PE and bleeding is low with or without LMWH (0–3 per 1000) [17].

Given that mechanical prophylaxis was previously shown to provide benefit compared to non-intervention [22,57], current VTE regimens within the ACCP guidelines suggest mechanical prophylaxis only, in the hope of reducing VTE risk without affecting bleeding risk. Despite this being common practice, evidence for mechanical prophylaxis in low VTE risk surgical patients is currently poor. A recent systematic review investigating GCS compared to no intervention failed to identify any such RCTs, identifying a single RCT arm, which compared the efficacy of GCS to LMWH in this patient demographic [58]. In this trial, 660 patients for knee arthroscopy received GCS, with 29/660 experiencing VTE (4.4%), which is higher than expected for a low VTE risk procedure [59]. Sub-group analysis revealed that those with meniscectomy were more likely to have VTE, which was possibly due to the longer immobility associated with the meniscal pathology.

Importantly, the extrapolation of data for mechanical prophylaxis from moderate to high VTE risk surgical patients is unlikely to be applicable to a low VTE risk group. The low incidence of VTE within the low VTE risk group may mean any potential reduction in VTE risk leads to a small absolute risk reduction in these patients, although it is noteworthy that procedures in this population represent very large numbers annually. Furthermore, lower risk patients are more likely to have minimally invasive and day-case surgery, resulting in nominal immobility. Given that mechanical prophylaxis is thought to counteract venous stasis associated with immobility, the reduction in VTE development may not be as apparent as in other patient groups who are immobile for longer periods of time post-operatively.

## 7. Future Clinical Practices in VTE Prevention for Surgical Patients

With a reduction in the number of VTE events occurring, future practice will likely utilise risk stratification to ensure that the appropriate VTE prophylaxis regimen is further tailored according to the risk of the patient.

For those deemed to be at low risk of VTE, prevention will likely consist of early mobilisation and locoregional anaesthesia as part of ERAS protocols without the need for mechanical prophylaxis. The use of GCS in this patient group is currently uncertain, but given the mounting evidence base questioning its efficacy in higher risk patients, its use may decline.

In patients deemed to be at medium or high risk of VTE, the use of pharmacological prophylaxis is justified if the risk of bleeding is not unacceptably high. Alternatives to LMWH are likely to emerge should appropriate clinical trials support, allowing easier administration of VTE prevention. DOACs have been validated within joint arthroplasty surgery. Ongoing trials are assessing whether aspirin is safe within orthopaedic patients and DOACs are safe within certain fields of surgery. The efficacy of IPC also remains unclear, with recent conflicting evidence emerging in the PREVENT trial, yet the trial by Kamachi et al. suggesting IPC to be a sufficient standalone intervention for VTE prevention [48,49]. However, it is likely to be used in conjunction with pharmacological prophylaxis in those with a particularly high risk or as a standalone intervention in those with a high risk of bleeding.

The use of GCS, in addition to pharmacological thromboprophylaxis, has recently been suggested to have no added benefit. Although GCS are largely safe, they can be associated with adverse events such as skin breaks and vascular compromise; hence, their use is in the balance in this context. Where mechanical prophylaxis may be required, such as those at high risk of bleeding or very high-risk patients, IPC will likely be selected over GCS, given that IPC is thought to be more effective.

## 8. Future Research in VTE Prevention for Surgical Patients

The development of RAMs for both risk of VTE and risk of bleeding would permit the ability for further personalisation of care. Such scores will need to be derived from a balanced population, so as to hold generalisability for wider use. Ideal scores would be both easy and quick to use, relying on factors elicited from a patient history. They would also require external validation in various patient groups to ensure their accuracy for clinical practice. However, it is important to be cautiously pessimistic in their development. 

Any validation study undertaken in a RAM will inherently be confounded by the indication for thromboprophylaxis. In order to assess their true predictive value, all participants would have to receive the same treatment to balance a therapeutic effect. There will never be ethical approval for a truly unbiased validation of a RAM either abstaining from or treating all with equal thromboprophylaxis, and hence, research in this area is plagued.

However, utilising such scores would also allow further stratification of patient populations, allowing trials to focus on more clinically similar patient groups. This may potentially counteract the overtreatment of those who may not require particular VTE interventions, allowing both a reduction in iatrogenic harm and healthcare cost savings.

Clinical data are additionally required to assess the role of mechanical prophylaxis. In particular, assessing the role of GCS in low VTE risk patients is particularly pertinent, due to its high national cost. Given the unit cost of stockings being £20.36 per patient, and there being ≈1 million operations compatible with being a day-case procedure within the NHS in England in the year 2018–2019, this would transpire to a cost of up to ≈£20 million per annum spent on GCS for patients deemed to be at low risk for VTE [60,61].

For IPC, its role within VTE prophylaxis regimens remains unclear. IPC is thought to have better efficacy than GCS, and so theoretically, it may hold use for patient groups at increased risk of VTE but for whom the risk of bleeding from pharmacological prophylaxis is not justifiable. In light of this rationale, ACCP guidelines advocate for an RCT investigating a comparison between pharmacological and IPC prophylaxis [17].

Initial evidence, despite being low quality, does suggest that NMES reduces the rates of DVT. However, NMES requires high-quality RCT data comparing it with existing regimens, including IPC and pharmacological prophylaxis of patients at various VTE risks. Cost analysis will also be needed to determine if NMES is cost-effective for widespread clinical practice.

Assessing the role of DOACs and aspirin outside of non-orthopaedic surgical patients is also needed to validate its wider use through well-constructed and appropriately powered RCTs. Such pharmacological prophylaxes are easy to administer, potentially improving compliance and cost-efficiency.

Future research may also investigate specific high VTE risk patient groups to allow for strong recommendations in such populations. For example, the ESA VTE task force identified surgery in obese patients as lacking in robust clinical evidence, with few RCTs identified in the literature and no RCTs outside of bariatric surgical patients, who are a fraction of the total obese surgical population [62].

Finally, whilst much emphasis is placed on determining pharmacological and mechanical regimens, improvement in the wider delivery of surgery has also improved VTE prevention. Researching into developing and applying systematic improvements will allow for a multi-faceted approach to preventing VTE.

## Figures and Tables

**Table 1:** Existing Risk Assessment Models (RAMs) development and validation.

Risk Assessment Model	Data Derived or Expert Derived Risk Factor Score Assignment	Validation			
		Internal or External Validation	Demographics	Sample Size	Accuracy
Department of Health, UK	NR	No validation study	-	-	-
Caprini	Expert	External	General, vascular, urology; ears, nose and throat; and critically ill surgical patients	8216; 2016; 4844	Higher risk groups significantly more likely to develop VTE [13,14,15]
Kucher	Expert	External	Acutely ill medical inpatients	63,458	Harrell’s c-index 0.563 (95% CI 0.558–0.568) [12]
Padua	Expert—adapted from Kucher	External	Acutely ill medical inpatients	63,458	Harrell’s c-index 0.600 (95% CI 0.594–0.606) [12]
Intermountain	Data	External	Acutely ill medical inpatients	63,458	Harrell’s c-index 0.611 (95% CI 0.605–0.618) [12]
International Medical Prevention Registry on Venous Thromboembolism (IMPROVE)	Data	External	Acutely ill medical inpatients	63,458	Harrell’s c-index 0.570 (95% CI 0.565–0.576) [12]
		External	Medical inpatients	19,217	ROC 0.70 [16]

CI—confidence interval; NR—not reported; ROC—Receiver Operator Characteristic.

**Table 2:** A summary of current ACCP guidelines for non-orthopaedic surgery [17].

(a)										
	Patient Group									
	Thoracic			Craniotomy		Spinal		Major Trauma		
**Risk of VTE**	Moderate	High	-	-	Very high (e.g., malignant disease)	-	High	-	High	-
**Risk of bleeding**	Not High	Low	High	-	Adequate haemostasis achieved	-	Adequate haemostasis achieved	-	-	CI to LMWH and LDUH
**Regimen**	Either MP or PP	Both MP and PP	MP only	MP only	Both MP and PP	MP only	Both MP and PP	Either MP or PP	Both MP and PP	MP only
**MP**	IPC (2C)	Either GCS or IPC (2C)	IPC preferred to GCS (2C)	IPC preferred to GCS (2C)	IPC preferred to GCS (2C)	IPC preferred to GCS (2C)	IPC preferred to GCS (2C)	IPC preferred to GCS (2C)	IPC preferred to GCS (2C)	IPC preferred to GCS(2C)
**PP**	Either LMWH or LDUH (2C)	Either LMWH or LDUH (1B)	Nil (2C)	Nil (2C)	No preference given (2C)	Nil (2C)	No preference given (2C)	Either LMWH or LDUH (2C)	No preference given (2C)	Add once no longer CI or risk reduces (2C)
**(b)**										
**Patient Group**										
	**General or Abdominal–Pelvic Surgery**							**Cardiac**		
**Risk of VTE**	Very Low	Low	Moderate	Moderate	High	High	High (Cancer)	Normal post-op course	Prolonged post-op course	
**Risk of bleeding**	-	-	Not High	High	Not High	High	Not High	-	-	
**Regimen**	Nil	MP only	Either MP or PP	MP only	Both MP and PP	MP only	PP only	MP only	Both MP and PP	
**MP**	Nil (2C)	IPC preferred to GCS (2C)	IPC preferred to GCS (2C)	IPC preferred to GCS (2C)	Either GCS or IPC (2C)	IPC preferred to GCS (2C)	-	IPC preferred to GCS (2C)	IPC preferred to GCS (2C)	
**PP**	Nil (1B)	-	Either LMWH or LDUH (2B)	-	Either LMWH or LDUH for 4 weeks (1B)	-	4 weeks LMWH (1B)	Nil (2C)	Either LDUH or LMWH (2C)	

The Grading of Recommendations Assessment, Development, and Evaluation (GRADE) score for each recommendation is noted in brackets within the table. VTE—venous thromboembolism; MP—mechanical prophylaxis; PP—pharmacological prophylaxis; LMWH—low molecular weight heparin; LDUH—low-dose unfractionated heparin; IPC—intermittent pneumatic compression; GCS—graduated compression stockings; CI—contraindications.

**Table 3:** ESA VTE guidelines for thromboprophylaxis in day-case or short-stay surgery [20].

	**VTE Risk**		
	Low patient and procedure risk	Either high patient or procedure risk	Both high patient and procedure risk
**Regimen**	No prophylaxis	LMWH ± IPC Orthopaedics: aspirin ± IPC	LMWH ± IPC

Adapted from Venclauskas et al. [20]. LMWH—low molecular weight heparin; IPC—intermittent pneumatic compression.

## References

[1] NHS Digital Deaths from Venous Thromboembolism (VTE) Related Events within 90 Days Post Discharge from Hospital. https://digital.nhs.uk/data-and-information/publications/statistical/nhs-outcomes-framework/february-2021/domain-5-treating-and-caring-for-people-in-a-safe-environment-and-protecting-them-from-avoidable-harm-nof/5.1-deaths-from-venous-thromboembolism-vte-related-events-within-90-days-post-discharge-from-hospital.

[2] Kahn S.R. (2016). The post-thrombotic syndrome. Hematol. Am. Soc. Hematol. Educ. Program.

[3] House of Commons Health Committee The Prevention of Venous Thromboembolism in Hospitalised Patients: Second Report of Session 2004–2005. https://publications.parliament.uk/pa/cm200405/cmselect/cmhealth/99/99.pdf.

[4] National Institute of Health and Care Excellence Venous Thromboembolism in over 16s: Reducing the Risk of Hospital-Acquired Deep Vein Thrombosis or Pulmonary Embolism, NICE Guideline [NG89]. https://www.nice.org.uk/guidance/ng89/chapter/Context.

[5] Anderson F.A., Spencer F.A. (2003). Risk Factors for Venous Thromboembolism. Circulation.

[6] Hunt B.J. (2019). Preventing hospital associated venous thromboembolism. BMJ.

[7] Thrombosis UK All-Part Parliamentary Thrombosis Group (APPTG) Survey Results 2019. https://thrombosisuk.org/downloads/APPTG%20Annual%20Review%202019%20100320.pdf.

[8] Machin M., Salim S., Onida S., Davies A.H. (2019). Venous thromboembolism risk assessment tools: Do we need a consensus?. Phlebology.

[9] Deheinzelin D., Braga A.L., Martins L.C., Martins M.A., Hernandez A., Yoshida W.B., Maffei F., Monachini M., Calderaro D., Campos W. (2006). Incorrect use of thromboprophylaxis for venous thromboembolism in medical and surgical patients: Results of a multicentric, observational and cross-sectional study in Brazil. J. Thromb. Haemost..

[10] Barbar S., Noventa F., Rossetto V., Ferrari A., Brandolin B., Perlati M., De Bon E., Tormene D., Pagnan A., Prandoni P. (2010). A risk assessment model for the identification of hospitalized medical patients at risk for venous thromboembolism: The Padua Prediction Score. J. Thromb. Haemost..

[11] Kucher N., Koo S., Quiroz R., Cooper J.M., Paterno M.D., Soukonnikov B., Goldhaber S.Z. (2005). Electronic alerts to prevent venous thromboembolism among hospitalized patients. N. Engl. J. Med..

[12] Greene M.T., Spyropoulos A.C., Chopra V., Grant P.J., Kaatz S., Bernstein S.J., Flanders S.A. (2016). Validation of Risk Assessment Models of Venous Thromboembolism in Hospitalized Medical Patients. Am. J. Med..

[13] Bahl V., Hu H.M., Henke P.K., Wakefield T.W., Campbell D.A., Caprini J.A. (2010). A validation study of a retrospective venous thromboembolism risk scoring method. Ann. Surg..

[14] Obi A.T., Pannucci C.J., Nackashi A., Abdullah N., Alvarez R., Bahl V., Wakefield T.W., Henke P.K. (2015). Validation of the Caprini Venous Thromboembolism Risk Assessment Model in Critically Ill Surgical Patients. JAMA Surg..

[15] Shuman A.G., Hu H.M., Pannucci C.J., Jackson C.R., Bradford C.R., Bahl V. (2012). Stratifying the risk of venous thromboembolism in otolaryngology. Otolaryngol. Head Neck Surg..

[16] Rosenberg D., Eichorn A., Alarcon M., McCullagh L., McGinn T., Spyropoulos A.C. (2014). External validation of the risk assessment model of the International Medical Prevention Registry on Venous Thromboembolism (IMPROVE) for medical patients in a tertiary health system. J. Am. Heart Assoc..

[17] Gould M.K., Garcia D.A., Wren S.M., Karanicolas P.J., Arcelus J.I., Heit J.A., Samama C.M. (2012). Prevention of VTE in nonorthopedic surgical patients: Antithrombotic Therapy and Prevention of Thrombosis, 9th ed: American College of Chest Physicians Evidence-Based Clinical Practice Guidelines. Chest.

[18] Falck-Ytter Y., Francis C.W., Johanson N.A., Curley C., Dahl O.E., Schulman S., Ortel T.L., Pauker S.G., Colwell C.W. (2012). Prevention of VTE in orthopedic surgery patients: Antithrombotic Therapy and Prevention of Thrombosis, 9th ed: American College of Chest Physicians Evidence-Based Clinical Practice Guidelines. Chest.

[19] Afshari A., Ageno W., Ahmed A., Duranteau J., Faraoni D., Kozek-Langenecker S., Llau J., Nizard J., Solca M., Stensballe J. (2018). European Guidelines on perioperative venous thromboembolism prophylaxis: Executive summary. Eur. J. Anaesthesiol. EJA.

[20] Venclauskas L., Llau J.V., Jenny J.Y., Kjaersgaard-Andersen P., Jans Ø. (2018). European guidelines on perioperative venous thromboembolism prophylaxis: Day surgery and fast-track surgery. Eur. J. Anaesthesiol..

[21] Afshari A., Fenger-Eriksen C., Monreal M., Verhamme P. (2018). European guidelines on perioperative venous thromboembolism prophylaxis: Mechanical prophylaxis. Eur. J. Anaesthesiol..

[22] Sachdeva A., Dalton M., Lees T. (2018). Graduated compression stockings for prevention of deep vein thrombosis. Cochrane Database Syst. Rev..

[23] Mismetti P., Laporte S., Darmon J.Y., Buchmüller A., Decousus H. (2001). Meta-analysis of low molecular weight heparin in the prevention of venous thromboembolism in general surgery. Br. J. Surg..

[24] Eppsteiner R.W., Shin J.J., Johnson J., van Dam R.M. (2010). Mechanical compression versus subcutaneous heparin therapy in postoperative and posttrauma patients: A systematic review and meta-analysis. World J. Surg..

[25] Turpie A.G.G., Lassen M.R., Davidson B.L., Bauer K.A., Gent M., Kwong L.M., Cushner F.D., Lotke P.A., Berkowitz S.D., Bandel T.J. (2009). Rivaroxaban versus enoxaparin for thromboprophylaxis after total knee arthroplasty (RECORD4): A randomised trial. Lancet.

[26] Eriksson B.I., Borris L.C., Friedman R.J., Haas S., Huisman M.V., Kakkar A.K., Bandel T.J., Beckmann H., Muehlhofer E., Misselwitz F. (2008). Rivaroxaban versus Enoxaparin for Thromboprophylaxis after Hip Arthroplasty. N. Engl. J. Med..

[27] Lassen M.R., Gallus A., Raskob G.E., Pineo G., Chen D., Ramirez L.M. (2010). Apixaban versus Enoxaparin for Thromboprophylaxis after Hip Replacement. N. Engl. J. Med..

[28] Lassen M.R., Raskob G.E., Gallus A., Pineo G., Chen D., Hornick P. (2010). Apixaban versus enoxaparin for thromboprophylaxis after knee replacement (ADVANCE-2): A randomised double-blind trial. Lancet.

[29] Eriksson B.I., Dahl O.E., Rosencher N., Kurth A.A., van Dijk C.N., Frostick S.P., Kälebo P., Christiansen A.V., Hantel S., Hettiarachchi R. (2007). Oral dabigatran etexilate vs. subcutaneous enoxaparin for the prevention of venous thromboembolism after total knee replacement: The RE-MODEL randomized trial. J. Thromb. Haemost..

[30] Eriksson B.I., Dahl O.E., Rosencher N., Kurth A.A., van Dijk C.N., Frostick S.P., Prins M.H., Hettiarachchi R., Hantel S., Schnee J. (2007). Dabigatran etexilate versus enoxaparin for prevention of venous thromboembolism after total hip replacement: A randomised, double-blind, non-inferiority trial. Lancet.

[31] Yu R., Nansubuga F., Yang J., Ding W., Li K., Weng D., Wu P., Chen G., Ma D., Wei J. (2020). Efficiency and safety evaluation of prophylaxes for venous thrombosis after gynecological surgery. Medicine.

[32] Clinical Trials.Gov Apixaban vs. Enoxaparin Following Microsurgical Breast Reconstruction-An RCT NCT04504318. NCT04504318.

[33] Clinical Trials.Gov The Direct Oral Anticoagulation Versus Warfarin After Cardiac Surgery Trial (DANCE) NCT04284839. NCT04284839.

[34] Becattini C., Pace U., Rondelli F., Delrio P., Ceccarelli G., Boncompagni M., Graziosi L., Visonà A., Chiari D., Avruscio G. (2020). Rivaroxaban for extended antithrombotic prophylaxis after laparoscopic surgery for colorectal cancer. Design of the PRO-LAPS II STUDY. Eur. J. Intern. Med..

[35] Kahn S.R., Shivakumar S. (2020). What’s new in VTE risk and prevention in orthopedic surgery. Res. Pract. Thromb. Haemost..

[36] Anderson D.R., Dunbar M., Murnaghan J., Kahn S.R., Gross P., Forsythe M., Pelet S., Fisher W., Belzile E., Dolan S. (2018). Aspirin or Rivaroxaban for VTE Prophylaxis after Hip or Knee Arthroplasty. N. Engl. J. Med..

[37] Pulmonary Embolism Prevention (PEP) Trial Collaborative Group (2000). Prevention of pulmonary embolism and deep vein thrombosis with low dose aspirin: Pulmonary Embolism Prevention (PEP) trial. Lancet.

[38] Matharu G.S., Kunutsor S.K., Judge A., Blom A.W., Whitehouse M.R. (2020). Clinical Effectiveness and Safety of Aspirin for Venous Thromboembolism Prophylaxis after Total Hip and Knee Replacement: A Systematic Review and Meta-analysis of Randomized Clinical Trials. JAMA Intern. Med..

[39] Clinical Trials.Gov Comparative Effectiveness of Pulmonary Embolism Prevention after Hip and Knee Replacement (PEPPER) NCT02810704. NCT02810704.

[40] Clincial Trials.Gov VTE Prevention Following Total Hip and Knee Arthroplasty (EPCATIII) NCT04075240. NCT04075240.

[41] Hamilton M.G., Yee W.H., Hull R.D., Ghali W.A. (2011). Venous Thromboembolism Prophylaxis in Patients Undergoing Cranial Neurosurgery: A Systematic Review and Meta-analysis. Neurosurgery.

[42] Mandavia R., Shalhoub J., Head K., Davies A.H. (2015). The additional benefit of graduated compression stockings to pharmacologic thromboprophylaxis in the prevention of venous thromboembolism in surgical inpatients. J. Vasc. Surg. Venous Lymphat. Disord..

[43] Cohen A.T., Skinner J.A., Warwick D., Brenkel I. (2007). The use of graduated compression stockings in association with fondaparinux in surgery of the hip. A multicentre, multinational, randomised, open-label, parallel-group comparative study. J. Bone Joint. Surg. Br..

[44] Shalhoub J., Lawton R., Hudson J., Baker C., Bradbury A., Dhillon K., Everington T., Gohel M.S., Hamady Z., Hunt B.J. (2020). Graduated compression stockings as adjuvant to pharmaco-thromboprophylaxis in elective surgical patients (GAPS study): Randomised controlled trial. BMJ.

[45] Suna K., Herrmann E., Kröger K., Schmandra T., Müller E., Hanisch E., Buia A. (2020). Graduated compression stockings in the prevention of postoperative pulmonary embolism. A propensity-matched retrospective case-control study of 24,273 patients. Ann. Med. Surg..

[46] The CLOTS Trial Collaboration (2009). Effectiveness of thigh-length graduated compression stockings to reduce the risk of deep vein thrombosis after stroke (CLOTS trial 1): A multicentre, randomised controlled trial. Lancet.

[47] Kakkos S.K., Caprini J.A., Geroulakos G., Nicolaides A.N., Stansby G., Reddy D.J., Ntouvas I. (2016). Combined intermittent pneumatic leg compression and pharmacological prophylaxis for prevention of venous thromboembolism. Cochrane Database Syst. Rev..

[48] Arabi Y.M., Al-Hameed F., Burns K.E.A., Mehta S., Alsolamy S.J., Alshahrani M.S., Mandourah Y., Almekhlafi G.A., Almaani M., Al Bshabshe A. (2019). Adjunctive Intermittent Pneumatic Compression for Venous Thromboprophylaxis. N. Engl. J. Med..

[49] Kamachi H., Homma S., Kawamura H., Yoshida T., Ohno Y., Ichikawa N., Yokota R., Funakoshi T., Maeda Y., Takahashi N. (2020). Intermittent pneumatic compression versus additional prophylaxis with enoxaparin for prevention of venous thromboembolism after laparoscopic surgery for gastric and colorectal malignancies: Multicentre randomized clinical trial. BJS Open.

[50] The CLOTS Trials Collaboration (2015). The Clots in Legs Or sTockings after Stroke (CLOTS) 3 trial: A randomised controlled trial to determine whether or not intermittent pneumatic compression reduces the risk of post-stroke deep vein thrombosis and to estimate its cost-effectiveness. Health Technol. Assess..

[51] Wang Y., Huang D., Wang M., Liang Z. (2020). Can Intermittent Pneumatic Compression Reduce the Incidence of Venous Thrombosis in Critically Ill Patients: A Systematic Review and Meta-Analysis. Clin. Appl. Thromb. Hemost..

[52] Ho K.M., Tan J.A. (2013). Stratified meta-analysis of intermittent pneumatic compression of the lower limbs to prevent venous thromboembolism in hospitalized patients. Circulation.

[53] Khatri A., Davies A.H., Shalhoub J. (2021). Mechanical prophylaxis for venous thromboembolism prevention in obese individuals. Phlebology.

[54] National Institute of Health and Care Excellence The Geko Device for Reducing the Risk of Venous Thromboembolism (MTG19). https://www.nice.org.uk/guidance/mtg19/resources/the-geko-device-for-reducing-the-risk-of-venous-thromboembolism-pdf-64371882777541.

[55] Ravikumar R., Williams K.J., Babber A., Moore H.M., Lane T.R., Shalhoub J., Davies A.H. (2018). Neuromuscular electrical stimulation for the prevention of venous thromboembolism. Phlebology.

[56] Hajibandeh S., Hajibandeh S., Antoniou G.A., Scurr J.R.H., Torella F. (2017). Neuromuscular electrical stimulation for the prevention of venous thromboembolism. Cochrane Database Syst. Rev..

[57] Urbankova J., Quiroz R., Kucher N., Goldhaber S.Z. (2005). Intermittent pneumatic compression and deep vein thrombosis prevention. A meta-analysis in postoperative patients. Thromb. Haemost..

[58] Machin M., Younan H.C., Smith S., Salim S., Davies A.H., Shalhoub J. (2021). Systematic review on the benefit of graduated compression stockings in the prevention of venous thromboembolism in low-risk surgical patients. Phlebology.

[59] Camporese G., Bernardi E., Prandoni P., Noventa F., Verlato F., Simioni P., Ntita K., Salmistraro G., Frangos C., Rossi F. (2008). Low-molecular-weight heparin versus compression stockings for thromboprophylaxis after knee arthroscopy: A randomized trial. Ann. Intern. Med..

[60] NHS Digital Hospital Admitted Patient Care Activity 2018-19. https://digital.nhs.uk/data-and-information/publications/statistical/hospital-admitted-patient-care-activity/2018-19.

[61] Shalhoub J., Norrie J., Baker C., Bradbury A.W., Dhillon K., Everington T., Gohel M.S., Hamady Z., Heatley F., Hudson J. (2017). Graduated Compression Stockings as an Adjunct to Low Dose Low Molecular Weight Heparin in Venous Thromboembolism Prevention in Surgery: A Multicentre Randomised Controlled Trial. Eur. J. Vasc. Endovasc. Surg..

[62] Venclauskas L., Maleckas A., Arcelus J.I., on behalf For the ESA VTE Guidelines Task Force on perioperative venous thromboembolism prophylaxis (2018). European guidelines on perioperative venous thromboembolism prophylaxis: Surgery in the obese patient. Eur. J. Anaesthesiol. EJA.

